# Correction: Rapid and robust antibody Fab fragment crystallization utilizing edge-to-edge beta-sheet packing

**DOI:** 10.1371/journal.pone.0258412

**Published:** 2021-10-05

**Authors:** The PLOS ONE Staff

The captions for Figs 5 and 6 are incorrectly switched. The publisher apologizes for the error. The caption that appears under Fig 5 should be under Fig 6, and the caption that appears under Fig 6 should be under Fig 5. The figure images appear in the correct order.

**Fig 5:**
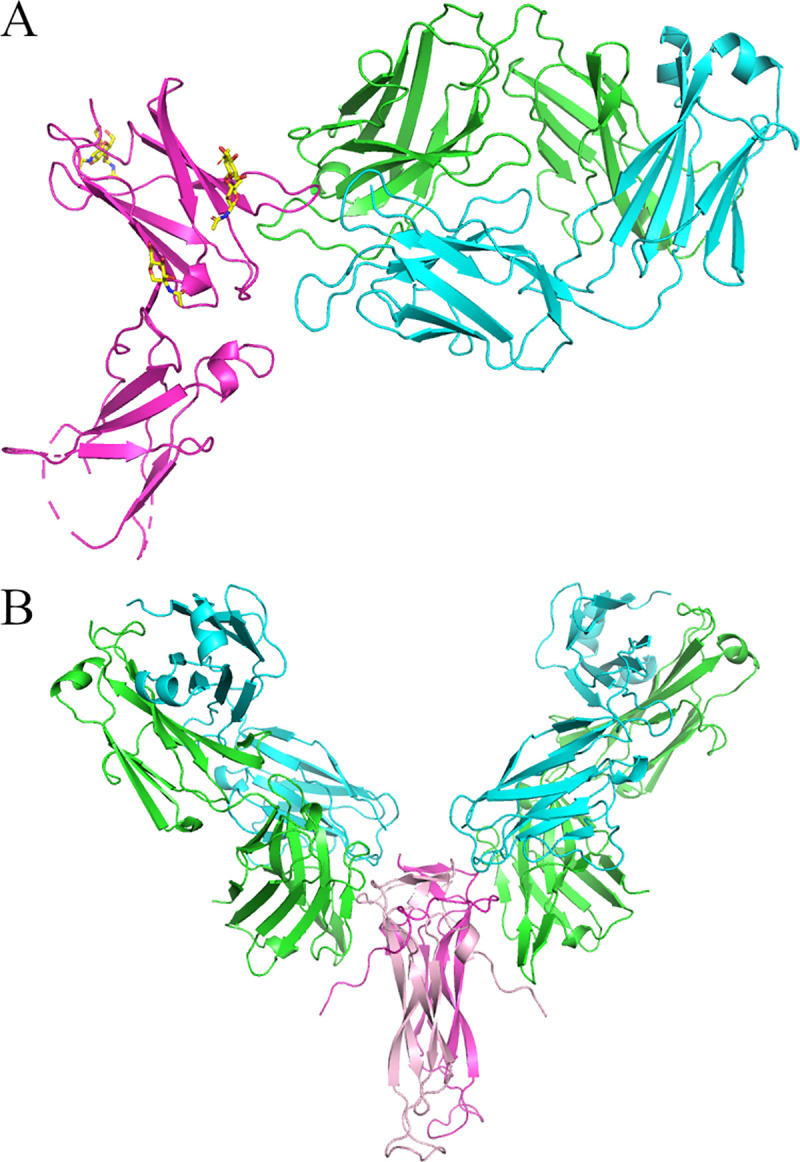
Crystal structures of Crystal Kappa variants in complexes. (A) Dupilumab Fab (HC in green and LC in cyan) complexed with human IL4R extracellular domain (in magenta and glycosylation in yellow sticks, PDB: 6WGL), (B) human IL17 dimer (magenta and pink) complexed with Secukinumab Fabs (HCs in green and LCs in cyan, PDB: 6WIR).

**Fig 6:**
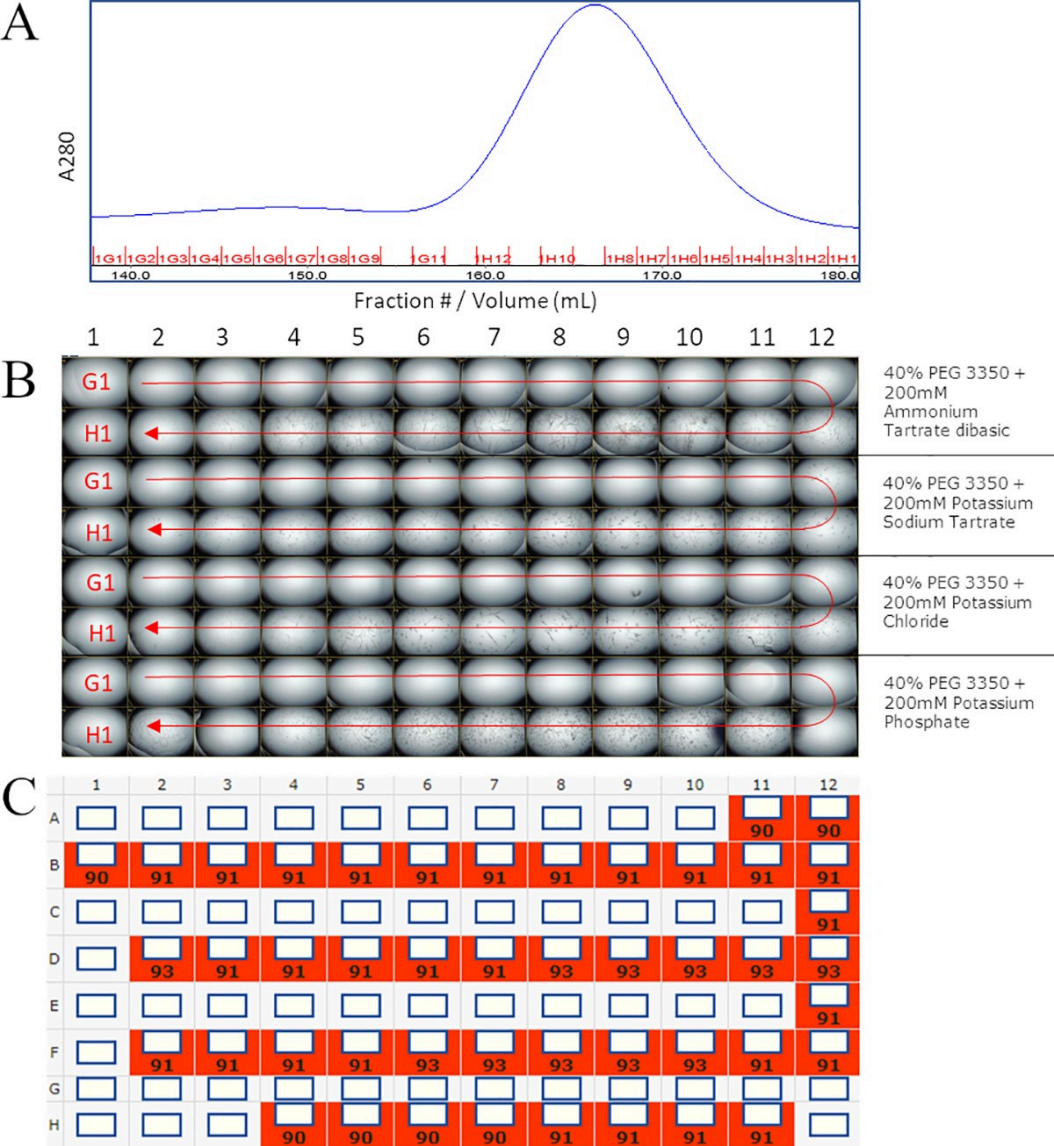
Column fraction crystallization. Column fractions from size exclusion chromatography were used directly in a vapor diffusion crystallization experiment in 4 conditions. (A) shows the chromatogram. (B) shows the images of every crystallization drop at day 9 (C) shows the score assigned to each, where anything crystalline is scored at 90 or higher.
