# Supplementary figures and images for: Rapid and robust antibody Fab fragment crystallization utilizing edge-to-edge beta-sheet packing
Source: PLoS One. 2020 Sep 11;15(9):e0232311. doi: 10.1371/journal.pone.0232311 (PMC7485759; doi:10.1371/journal.pone.0232311)

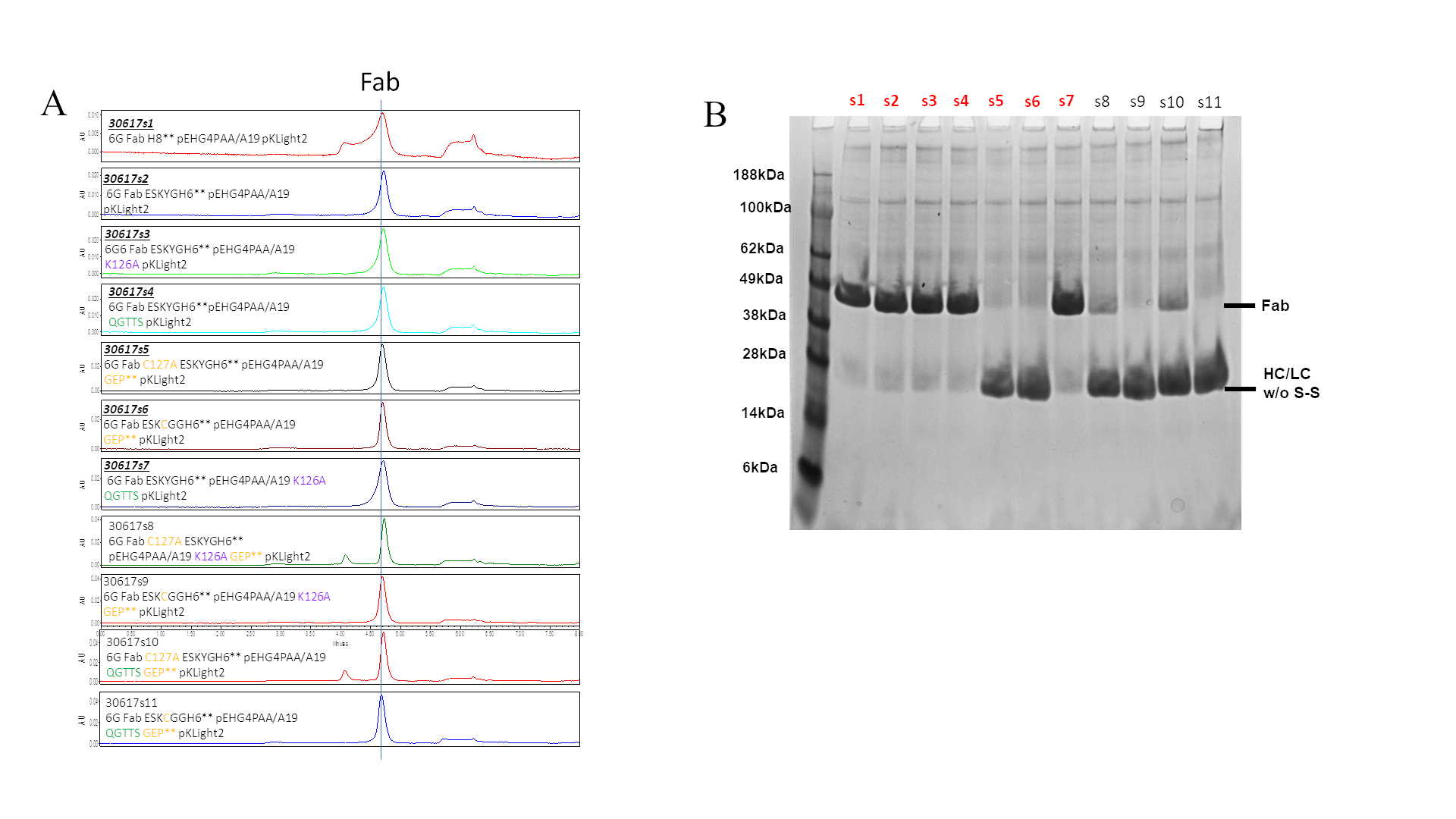

Supplement: S1 Fig — Shows well-formed Fabs. A) Fabs are expression and IMAC purified. aSEC shows fabs are well formed. B) Non-reduced SDS-PAGE gel shows Fabs are intacted. (TIF) [file pone.0232311.s001.tif]

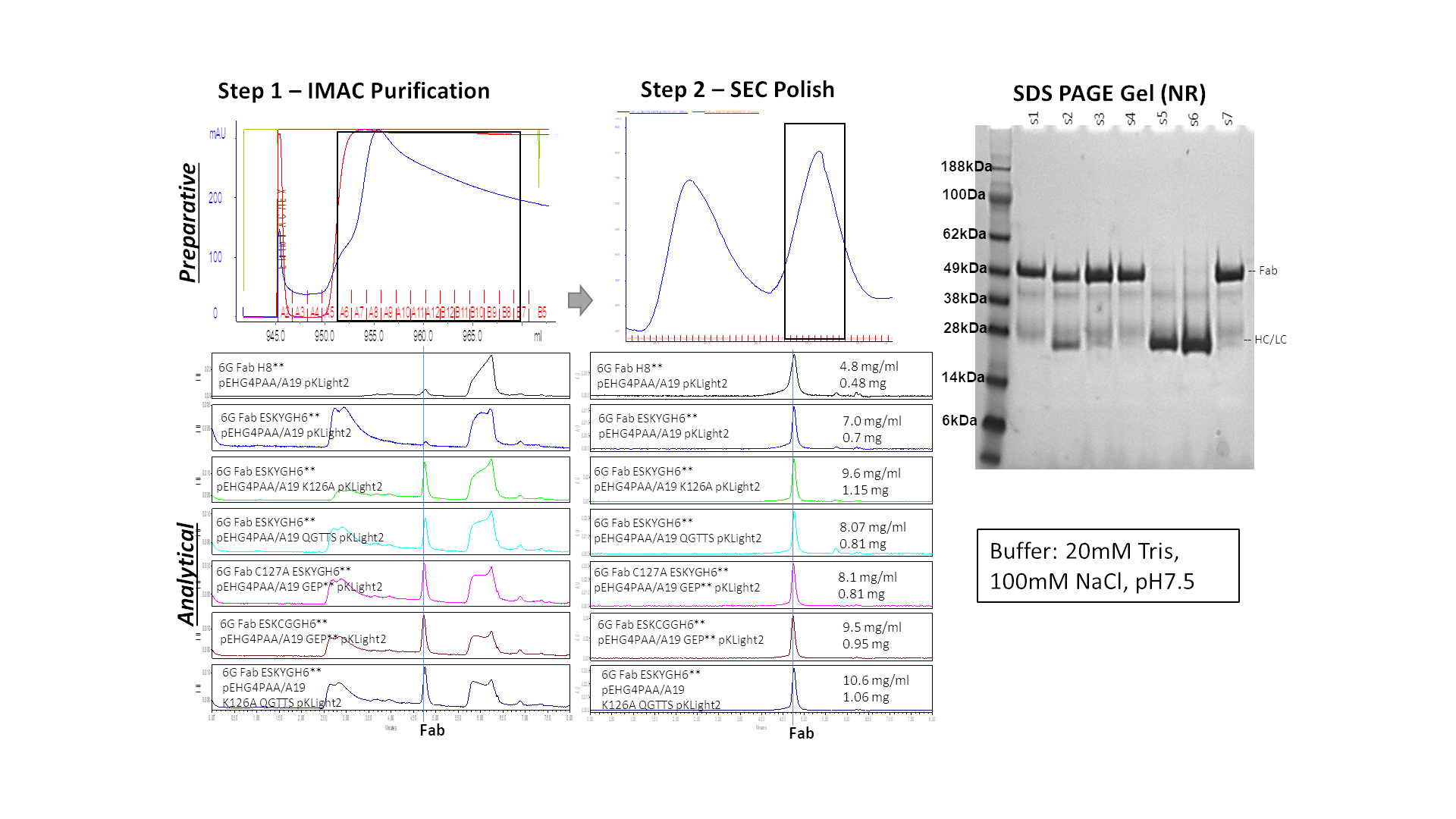

Supplement: S2 Fig — (TIF) [file pone.0232311.s002.tif]
