# Supplementary material for: Rapid and robust antibody Fab fragment crystallization utilizing edge-to-edge beta-sheet packing
Source: PLoS One. 2020 Sep 11;15(9):e0232311. doi: 10.1371/journal.pone.0232311 (PMC7485759; doi:10.1371/journal.pone.0232311)
Supplement: S1 Table — (DOCX) [file pone.0232311.s003.docx]

| PDB | Parental Antibody | Isotype | CK | HC variant | LC variant | Resolution |
| --- | --- | --- | --- | --- | --- | --- |
| **6WGB** | Dupilumab | IgG4 | parental | wild-type | wild-type | 1.99 |
| **6WG8** | Dupilumab | IgG4 | CK1.0 |  | ∆QGTTS∆ | 1.36 |
| **6WGJ** | Dupilumab | IgG4 | CK1.2 | C127A | ∆QGTTS∆ GEP* | 1.90 |
| **6WGK** | Dupilumab | IgG4 | CK1.3 | ESKCGGH6 | ∆QGTTS∆ GEP* | 1.62 |
| **6WGL** | Dupilumab:hIL4R | IgG4 | CK1.0 |  | ∆QGTTS∆ | 2.82 |
| **6WIR** | Secukinumab:hIL17 | IgG4 | CK1.0 |  | ∆QGTTS∆ | 3.00 |
| **6WIO** | Secukinumab:hIL17 | IgG1 | CK3.3 | KSC* | ∆QGTTS∆ | 2.40 |
