# Supplementary material for: Rapid and robust antibody Fab fragment crystallization utilizing edge-to-edge beta-sheet packing
Source: PLoS One. 2020 Sep 11;15(9):e0232311. doi: 10.1371/journal.pone.0232311 (PMC7485759; doi:10.1371/journal.pone.0232311)
Supplement: S2 File — (PDF) [file pone.0232311.s005.pdf]

Full wwPDB X-ray Structure Validation Report (i)

Apr 6, 2020 – 02:39 PM EDT

PDB ID	:	6WGB
Title	:	A Fab domain of Dupilumab
Deposited on	:	2020-04-05
Resolution	:	1.99 Å(reported)

This is a Full wwPDB X-ray Structure Validation Report.

This report is produced by the wwPDB biocuration pipeline after annotation of the structure.

We welcome your comments at *validation@mail.wwpdb.org*
A user guide is available at
https://www.wwpdb.org/validation/2017/XrayValidationReportHelp
with specific help available everywhere you see the (i) symbol.

CONFIDENTIAL VALIDATION REPORT

The following versions of software and data (see references (i)) were used in the production of this report:

MolProbity	:	4.02b-467
Xtriage (Phenix)	:	1.13
EDS	:	2.10.1
Percentile statistics	:	20171227.v01 (using entries in the PDB archive December 27th 2017)
Refmac	:	5.8.0158
CCP4	:	7.0.044 (Gargrove)
Ideal geometry (proteins)	:	Engh & Huber (2001)
Ideal geometry (DNA, RNA)	:	Parkinson et al. (1996)
Validation Pipeline (wwPDB-VP)	:	2.10.1

1 Overall quality at a glance (i)

The following experimental techniques were used to determine the structure:
X-RAY DIFFRACTION

The reported resolution of this entry is 1.99 Å.

Percentile scores (ranging between 0-100) for global validation metrics of the entry are shown in the following graphic. The table shows the number of entries on which the scores are based.

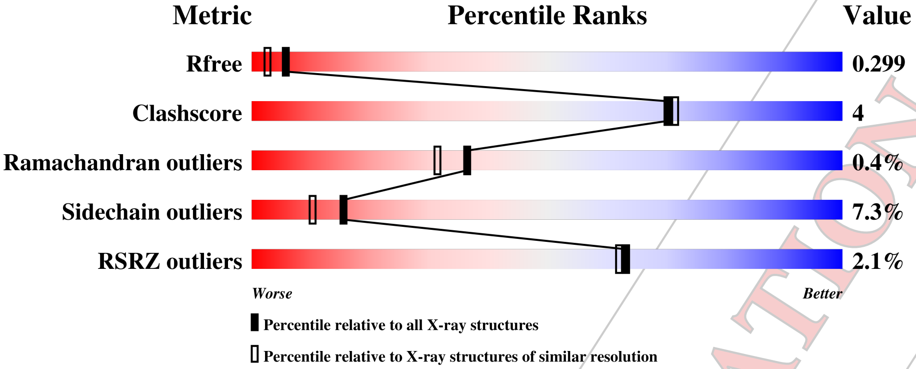

Metric	Whole archive (#Entries)	Similar resolution (#Entries, resolution range(Å))
R_{free}	111664	7193 (2.00-2.00)
Clashscore	122126	8267 (2.00-2.00)
Ramachandran outliers	120053	8166 (2.00-2.00)
Sidechain outliers	120020	8165 (2.00-2.00)
RSRZ outliers	108989	7011 (2.00-2.00)

The table below summarises the geometric issues observed across the polymeric chains and their fit to the electron density. The red, orange, yellow and green segments on the lower bar indicate the fraction of residues that contain outliers for >=3, 2, 1 and 0 types of geometric quality criteria respectively. A grey segment represents the fraction of residues that are not modelled. The numeric value for each fraction is indicated below the corresponding segment, with a dot representing fractions <=5% The upper red bar (where present) indicates the fraction of residues that have poor fit to the electron density. The numeric value is given above the bar.

Mol	Chain	Length	Quality of chain
1	A	234	78% 10% • 11%
1	C	234	5% / 72% 10% • 15%
2	B	219	85% 12% • •
2	D	219	2% / 78% 13% • 5%

CONFIDENTIAL VALIDATION REPORT

2 Entry composition (i)

There are 3 unique types of molecules in this entry. The entry contains 6179 atoms, of which 0 are hydrogens and 0 are deuteriums.

In the tables below, the ZeroOcc column contains the number of atoms modelled with zero occupancy, the AltConf column contains the number of residues with at least one atom in alternate conformation and the Trace column contains the number of residues modelled with at most 2 atoms.

- Molecule 1 is a protein called Dupilumab Fab heavy chain.

Mol	Chain	Residues	Atoms	ZeroOcc	AltConf	Trace
1	A	208	Total C N O S<br>1519 953 256 303 7	0	0	0
1	C	198	Total C N O S<br>1388 873 234 275 6	0	0	0

- Molecule 2 is a protein called Dupilumab Fab light chain.

Mol	Chain	Residues	Atoms	ZeroOcc	AltConf	Trace
2	B	215	Total C N O S<br>1627 1024 268 329 6	0	0	0
2	D	208	Total C N O S<br>1528 955 254 313 6	0	0	0

- Molecule 3 is water.

Mol	Chain	Residues	Atoms	ZeroOcc	AltConf
3	A	38	Total O<br>38 38	0	0
3	B	57	Total O<br>57 57	0	0
3	C	8	Total O<br>8 8	0	0
3	D	14	Total O<br>14 14	0	0

CONFIDENTIAL VALIDATION REPORT

3 Residue-property plots (i)

These plots are drawn for all protein, RNA and DNA chains in the entry. The first graphic for a chain summarises the proportions of the various outlier classes displayed in the second graphic. The second graphic shows the sequence view annotated by issues in geometry and electron density. Residues are color-coded according to the number of geometric quality criteria for which they contain at least one outlier: green = 0, yellow = 1, orange = 2 and red = 3 or more. A red dot above a residue indicates a poor fit to the electron density (RSRZ > 2). Stretches of 2 or more consecutive residues without any outlier are shown as a green connector. Residues present in the sample, but not in the model, are shown in grey.

- Molecule 1: Dupilumab Fab heavy chain

Chain A:

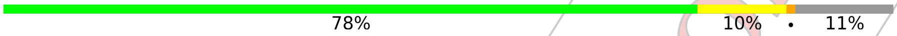

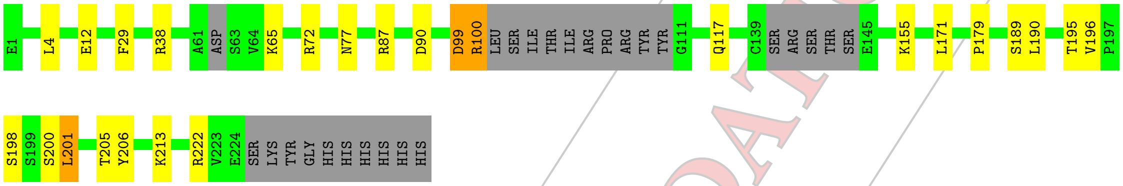

- Molecule 1: Dupilumab Fab heavy chain

Chain C:

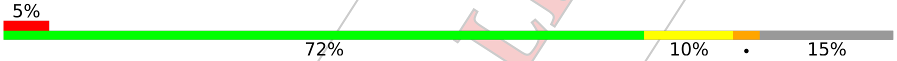

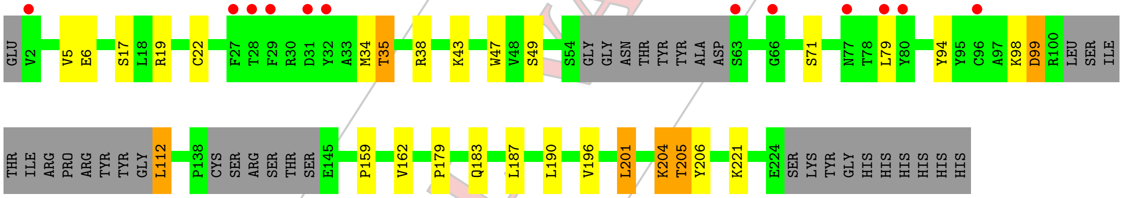

- Molecule 2: Dupilumab Fab light chain

Chain B:

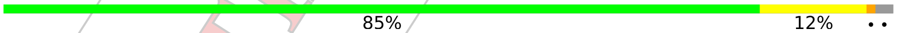

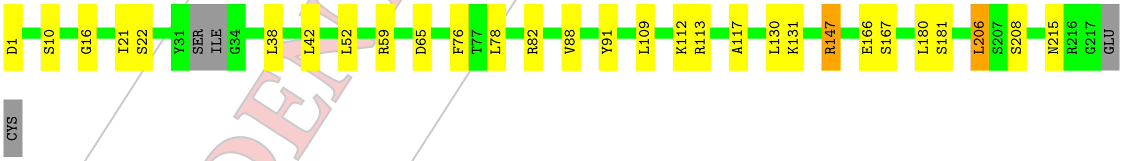

- Molecule 2: Dupilumab Fab light chain

Chain D:

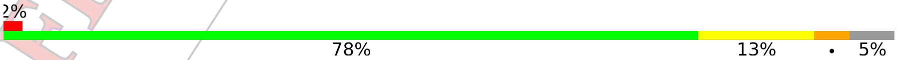

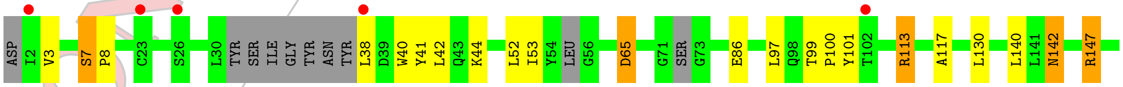

CONFIDENTIAL VALIDATION REPORT

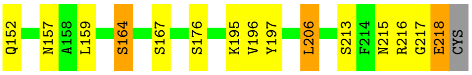

CONFIDENTIAL VALIDATION REPORT

4 Data and refinement statistics (i)

Property	Value	Source
Space group	P 1 21 1	Depositor
Cell constants<br>a, b, c, α, β, γ	52.75Å 66.29Å 135.48Å<br>90.00° 91.64° 90.00°	Depositor
Resolution (Å)	30.00 – 1.99<br>67.71 – 1.99	Depositor<br>EDS
% Data completeness<br>(in resolution range)	99.6 (30.00-1.99)<br>99.6 (67.71-1.99)	Depositor<br>EDS
R_{merge}	0.06	Depositor
R_{sym}	(Not available)	Depositor
$< I/\sigma(I) >$ [1]	2.06 (at 1.98Å)	Xtriage
Refinement program	REFMAC 5.8.0103	Depositor
R, R_{free}	0.243 , 0.299<br>0.244 , 0.299	Depositor<br>DCC
R_{free} test set	3098 reflections (4.84%)	wwPDB-VP
Wilson B-factor (Å^2)	29.4	Xtriage
Anisotropy	0.093	Xtriage
Bulk solvent k_{sol}(e/Å^3), B_{sol}(Å^2)	0.33 , 22.3	EDS
L-test for twinning[2]	$< \|L\| > = 0.48$, $< L^2 > = 0.31$	Xtriage
Estimated twinning fraction	0.125 for h,-k,-l	Xtriage
F_o,F_c correlation	0.93	EDS
Total number of atoms	6179	wwPDB-VP
Average B, all atoms (Å^2)	33.0	wwPDB-VP

Xtriage's analysis on translational NCS is as follows: *The largest off-origin peak in the Patterson function is 5.85% of the height of the origin peak. No significant pseudotranslation is detected.*

[1] Intensities estimated from amplitudes.

[2] Theoretical values of $< |L| >$, $< L^2 >$ for acentric reflections are 0.5, 0.333 respectively for untwinned datasets, and 0.375, 0.2 for perfectly twinned datasets.

CONFIDENTIAL VALIDATION REPORT

5 Model quality (i)

5.1 Standard geometry (i)

The Z score for a bond length (or angle) is the number of standard deviations the observed value is removed from the expected value. A bond length (or angle) with $|Z| > 5$ is considered an outlier worth inspection. RMSZ is the root-mean-square of all Z scores of the bond lengths (or angles).

Mol	Chain	Bond lengths		Bond angles	
		RMSZ	$\#\|Z\| > 5$	RMSZ	$\#\|Z\| > 5$
1	A	0.50	0/1550	0.93	2/2110 (0.1%)
1	C	0.47	0/1416	0.94	1/1935 (0.1%)
2	B	0.51	0/1663	0.92	1/2263 (0.0%)
2	D	0.47	0/1559	0.90	0/2124
All	All	0.49	0/6188	0.92	4/8432 (0.0%)

Chiral center outliers are detected by calculating the chiral volume of a chiral center and verifying if the center is modelled as a planar moiety or with the opposite hand.A planarity outlier is detected by checking planarity of atoms in a peptide group, atoms in a mainchain group or atoms of a sidechain that are expected to be planar.

Mol	Chain	#Chirality outliers	#Planarity outliers
1	A	0	3
1	C	0	2
2	B	0	2
2	D	0	3
All	All	0	10

There are no bond length outliers.

All (4) bond angle outliers are listed below:

Mol	Chain	Res	Type	Atoms	Z	Observed(^{o})	Ideal(^{o})
2	B	166	GLU	CB-CA-C	-6.44	97.52	110.40
1	C	205	THR	CA-CB-OG1	-5.94	96.53	109.00
1	A	195	THR	CA-CB-OG1	-5.56	97.33	109.00
1	A	99	ASP	CB-CA-C	5.16	120.72	110.40

There are no chirality outliers.

All (10) planarity outliers are listed below:

Mol	Chain	Res	Type	Group
1	A	222	ARG	Sidechain

Continued on next page...

CONFIDENTIAL VALIDATION REPORT

Continued from previous page...

Mol	Chain	Res	Type	Group
1	A	38	ARG	Sidechain
1	A	72	ARG	Sidechain
2	B	113	ARG	Sidechain
2	B	147	ARG	Sidechain
1	C	19	ARG	Sidechain
1	C	38	ARG	Sidechain
2	D	147	ARG	Sidechain
2	D	216	ARG	Sidechain
2	D	99	THR	Peptide

5.2 Too-close contacts (i)

In the following table, the Non-H and H(model) columns list the number of non-hydrogen atoms and hydrogen atoms in the chain respectively. The H(added) column lists the number of hydrogen atoms added and optimized by MolProbity. The Clashes column lists the number of clashes within the asymmetric unit, whereas Symm-Clashes lists symmetry related clashes.

Mol	Chain	Non-H	H(model)	H(added)	Clashes	Symm-Clashes
1	A	1519	0	1454	9	0
1	C	1388	0	1268	14	0
2	B	1627	0	1548	9	0
2	D	1528	0	1395	18	0
3	A	38	0	0	1	0
3	B	57	0	0	1	0
3	C	8	0	0	0	0
3	D	14	0	0	1	0
All	All	6179	0	5665	46	0

The all-atom clashscore is defined as the number of clashes found per 1000 atoms (including hydrogen atoms). The all-atom clashscore for this structure is 4.

All (46) close contacts within the same asymmetric unit are listed below, sorted by their clash magnitude.

Atom-1	Atom-2	Interatomic distance (Å)	Clash overlap (Å)
1:A:205:THR:HG22	3:A:301:HOH:O	1.77	0.83
2:D:140:LEU:HD21	2:D:142:ASN:OD1	1.97	0.65
1:C:98:LYS:O	1:C:112:LEU:HD12	2.00	0.60
1:C:112:LEU:N	2:D:41:TYR:HH	1.99	0.60
2:D:152:GLN:HG3	2:D:159:LEU:CD1	2.33	0.59
2:D:164:SER:HB2	3:D:307:HOH:O	2.04	0.57

Continued on next page...

CONFIDENTIAL VALIDATION REPORT

Continued from previous page...

Atom-1	Atom-2	Interatomic distance (Å)	Clash overlap (Å)
2:D:196:VAL:HG22	2:D:215:ASN:HD22	1.70	0.56
2:B:16:GLY:HA2	2:B:82:ARG:HG3	1.88	0.56
1:A:155:LYS:HG3	1:A:189:SER:OG	2.06	0.55
2:B:21:ILE:HD12	2:B:78:LEU:HD23	1.88	0.54
1:C:47:TRP:CZ3	2:D:100:PRO:HB3	2.42	0.54
2:B:38:LEU:HD13	2:B:76:PHE:CD1	2.43	0.54
2:D:217:GLY:O	2:D:218:GLU:HG3	2.07	0.54
2:D:197:TYR:O	2:D:213:SER:HA	2.08	0.53
2:D:42:LEU:HD21	2:D:44:LYS:HE2	1.90	0.52
1:C:71:SER:O	1:C:79:LEU:HD12	2.10	0.52
2:D:113:ARG:HG3	2:D:176:SER:HB2	1.92	0.50
1:C:6:GLU:OE2	1:C:94:TYR:O	2.29	0.50
2:D:101:TYR:N	2:D:101:TYR:CD1	2.80	0.49
2:D:40:TRP:HB2	2:D:53:ILE:HB	1.94	0.49
2:D:117:ALA:HB1	2:D:206:LEU:HD13	1.94	0.49
1:C:35:THR:HG23	1:C:99:ASP:OD1	2.14	0.48
1:C:183:GLN:HG3	1:C:187:LEU:O	2.14	0.48
2:D:152:GLN:HG3	2:D:159:LEU:HD13	1.96	0.47
2:D:195:LYS:O	2:D:215:ASN:HA	2.14	0.47
1:C:5:VAL:O	1:C:22:CYS:HA	2.15	0.47
1:C:34:MET:HB3	1:C:79:LEU:HD22	1.97	0.47
2:B:59:ARG:NH1	3:B:301:HOH:O	2.48	0.47
2:B:42:LEU:HD13	2:B:91:TYR:CZ	2.50	0.47
1:A:87:ARG:O	1:A:90:ASP:HB2	2.15	0.46
1:A:198:SER:O	1:A:201:LEU:HB2	2.16	0.46
1:C:196:VAL:HG11	1:C:206:TYR:CE1	2.51	0.46
1:A:99:ASP:OD1	1:A:100:ARG:N	2.42	0.45
1:C:179:PRO:HD2	2:D:167:SER:OG	2.18	0.43
1:A:190:LEU:C	1:A:190:LEU:HD12	2.39	0.43
1:A:29:PHE:CD2	1:A:77:ASN:HA	2.53	0.43
2:B:88:VAL:HG23	2:B:109:LEU:O	2.18	0.43
1:C:201:LEU:HA	1:C:201:LEU:HD12	1.92	0.42
1:A:179:PRO:HD2	2:B:167:SER:OG	2.19	0.42
1:A:196:VAL:HG11	1:A:206:TYR:CE1	2.55	0.41
2:D:7:SER:HA	2:D:8:PRO:HA	1.84	0.41
2:B:117:ALA:HB1	2:B:206:LEU:HD13	2.03	0.41
1:C:190:LEU:HD12	1:C:190:LEU:C	2.41	0.41
2:D:152:GLN:CG	2:D:159:LEU:HD13	2.50	0.41
1:C:204:LYS:HD2	1:C:206:TYR:CZ	2.56	0.40
2:B:180:LEU:C	2:B:180:LEU:HD23	2.42	0.40

There are no symmetry-related clashes.

CONFIDENTIAL VALIDATION REPORT

5.3 Torsion angles (i)

5.3.1 Protein backbone (i)

In the following table, the Percentiles column shows the percent Ramachandran outliers of the chain as a percentile score with respect to all X-ray entries followed by that with respect to entries of similar resolution.

The Analysed column shows the number of residues for which the backbone conformation was analysed, and the total number of residues.

Mol	Chain	Analysed	Favoured	Allowed	Outliers	Percentiles	
1	A	200/234 (86%)	195 (98%)	4 (2%)	1 (0%)	31	25
1	C	190/234 (81%)	178 (94%)	12 (6%)	0	100	100
2	B	211/219 (96%)	205 (97%)	6 (3%)	0	100	100
2	D	200/219 (91%)	190 (95%)	8 (4%)	2 (1%)	17	10
All	All	801/906 (88%)	768 (96%)	30 (4%)	3 (0%)	36	31

All (3) Ramachandran outliers are listed below:

Mol	Chain	Res	Type
2	D	65	ASP
2	D	97	LEU
1	A	65	LYS

5.3.2 Protein sidechains (i)

In the following table, the Percentiles column shows the percent sidechain outliers of the chain as a percentile score with respect to all X-ray entries followed by that with respect to entries of similar resolution.

The Analysed column shows the number of residues for which the sidechain conformation was analysed, and the total number of residues.

Mol	Chain	Analysed	Rotameric	Outliers	Percentiles	
1	A	165/198 (83%)	157 (95%)	8 (5%)	28	24
1	C	139/198 (70%)	127 (91%)	12 (9%)	11	7
2	B	181/193 (94%)	168 (93%)	13 (7%)	16	10
2	D	163/193 (84%)	149 (91%)	14 (9%)	11	7
All	All	648/782 (83%)	601 (93%)	47 (7%)	15	10

All (47) residues with a non-rotameric sidechain are listed below:

CONFIDENTIAL VALIDATION REPORT

Mol	Chain	Res	Type
1	A	4	LEU
1	A	12	GLU
1	A	100	ARG
1	A	117	GLN
1	A	171	LEU
1	A	200	SER
1	A	201	LEU
1	A	213	LYS
2	B	1	ASP
2	B	10	SER
2	B	22	SER
2	B	52	LEU
2	B	65	ASP
2	B	112	LYS
2	B	130	LEU
2	B	131	LYS
2	B	147	ARG
2	B	181	SER
2	B	206	LEU
2	B	208	SER
2	B	215	ASN
1	C	17	SER
1	C	35	THR
1	C	43	LYS
1	C	49	SER
1	C	99	ASP
1	C	112	LEU
1	C	159	PRO
1	C	162	VAL
1	C	201	LEU
1	C	204	LYS
1	C	205	THR
1	C	221	LYS
2	D	3	VAL
2	D	7	SER
2	D	38	LEU
2	D	52	LEU
2	D	65	ASP
2	D	86	GLU
2	D	113	ARG
2	D	130	LEU
2	D	142	ASN
2	D	147	ARG

Continued on next page...

CONFIDENTIAL VALIDATION REPORT

Continued from previous page...

Mol	Chain	Res	Type
2	D	157	ASN
2	D	164	SER
2	D	206	LEU
2	D	218	GLU

Some sidechains can be flipped to improve hydrogen bonding and reduce clashes. All (1) such sidechains are listed below:

Mol	Chain	Res	Type
2	D	215	ASN

5.3.3 RNA (i)

There are no RNA molecules in this entry.

5.4 Non-standard residues in protein, DNA, RNA chains (i)

There are no non-standard protein/DNA/RNA residues in this entry.

5.5 Carbohydrates (i)

There are no carbohydrates in this entry.

5.6 Ligand geometry (i)

There are no ligands in this entry.

5.7 Other polymers (i)

There are no such residues in this entry.

5.8 Polymer linkage issues (i)

There are no chain breaks in this entry.

CONFIDENTIAL VALIDATION REPORT

6 Fit of model and data (i)

6.1 Protein, DNA and RNA chains (i)

In the following table, the column labelled '#RSRZ> 2' contains the number (and percentage) of RSRZ outliers, followed by percent RSRZ outliers for the chain as percentile scores relative to all X-ray entries and entries of similar resolution. The OWAB column contains the minimum, median, 95th percentile and maximum values of the occupancy-weighted average B-factor per residue. The column labelled 'Q< 0.9' lists the number of (and percentage) of residues with an average occupancy less than 0.9.

Mol	Chain	Analysed	<RSRZ>	#RSRZ>2			OWAB(Å^2)	Q<0.9
1	A	208/234 (88%)	-0.11	0	100	100	16, 29, 44, 59	0
1	C	198/234 (84%)	0.30	12 (6%)	21	20	18, 37, 64, 79	0
2	B	215/219 (98%)	-0.13	0	100	100	18, 29, 47, 56	0
2	D	208/219 (94%)	0.13	5 (2%)	59	58	18, 33, 61, 82	0
All	All	829/906 (91%)	0.04	17 (2%)	63	62	16, 32, 57, 82	0

All (17) RSRZ outliers are listed below:

Mol	Chain	Res	Type	RSRZ
1	C	66	GLY	3.2
1	C	29	PHE	3.1
1	C	80	TYR	3.1
1	C	27	PHE	3.1
2	D	2	ILE	3.0
1	C	31	ASP	2.5
2	D	26	SER	2.5
1	C	2	VAL	2.4
1	C	32	TYR	2.4
1	C	28	THR	2.4
2	D	38	LEU	2.3
1	C	63	SER	2.2
1	C	79	LEU	2.1
2	D	102	THR	2.0
1	C	96	CYS	2.0
1	C	77	ASN	2.0
2	D	23	CYS	2.0

6.2 Non-standard residues in protein, DNA, RNA chains (i)

There are no non-standard protein/DNA/RNA residues in this entry.

CONFIDENTIAL VALIDATION REPORT

6.3 Carbohydrates (i)

There are no carbohydrates in this entry.

6.4 Ligands (i)

There are no ligands in this entry.

6.5 Other polymers (i)

There are no such residues in this entry.

CONFIDENTIAL VALIDATION REPORT
