# Supplementary material for: Rapid and robust antibody Fab fragment crystallization utilizing edge-to-edge beta-sheet packing
Source: PLoS One. 2020 Sep 11;15(9):e0232311. doi: 10.1371/journal.pone.0232311 (PMC7485759; doi:10.1371/journal.pone.0232311)
Supplement: S4 File — (PDF) [file pone.0232311.s007.pdf]

Full wwPDB X-ray Structure Validation Report (i)

Apr 6, 2020 – 02:44 PM EDT

PDB ID	:	6WGK
Title	:	A Fab derived from Dupilumab
Deposited on	:	2020-04-05
Resolution	:	1.62 Å(reported)

This is a Full wwPDB X-ray Structure Validation Report.

This report is produced by the wwPDB biocuration pipeline after annotation of the structure.

We welcome your comments at *validation@mail.wwpdb.org*
A user guide is available at
https://www.wwpdb.org/validation/2017/XrayValidationReportHelp
with specific help available everywhere you see the (i) symbol.

The following versions of software and data (see references (i)) were used in the production of this report:

MolProbity	:	4.02b-467
Xtriage (Phenix)	:	1.13
EDS	:	2.10.1
Percentile statistics	:	20171227.v01 (using entries in the PDB archive December 27th 2017)
Refmac	:	5.8.0158
CCP4	:	7.0.044 (Gargrove)
Ideal geometry (proteins)	:	Engh & Huber (2001)
Ideal geometry (DNA, RNA)	:	Parkinson et al. (1996)
Validation Pipeline (wwPDB-VP)	:	2.10.1

CONFIDENTIAL VALIDATION REPORT

1 Overall quality at a glance (i)

The following experimental techniques were used to determine the structure:
X-RAY DIFFRACTION

The reported resolution of this entry is 1.62 Å.

Percentile scores (ranging between 0-100) for global validation metrics of the entry are shown in the following graphic. The table shows the number of entries on which the scores are based.

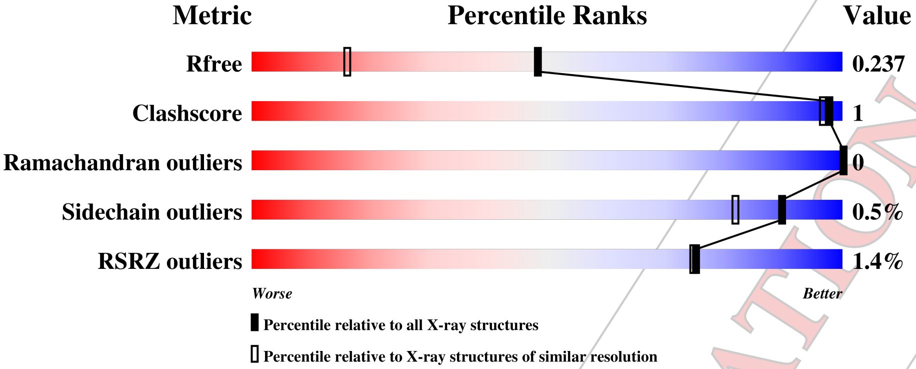

Metric	Whole archive (#Entries)	Similar resolution (#Entries, resolution range(Å))
R_{free}	111664	3975 (1.64-1.60)
Clashscore	122126	4258 (1.64-1.60)
Ramachandran outliers	120053	4162 (1.64-1.60)
Sidechain outliers	120020	4161 (1.64-1.60)
RSRZ outliers	108989	3894 (1.64-1.60)

The table below summarises the geometric issues observed across the polymeric chains and their fit to the electron density. The red, orange, yellow and green segments on the lower bar indicate the fraction of residues that contain outliers for >=3, 2, 1 and 0 types of geometric quality criteria respectively. A grey segment represents the fraction of residues that are not modelled. The numeric value for each fraction is indicated below the corresponding segment, with a dot representing fractions <=5% The upper red bar (where present) indicates the fraction of residues that have poor fit to the electron density. The numeric value is given above the bar.

Mol	Chain	Length	Quality of chain
1	A	235	2%<br>89% • 7%
1	C	235	3%<br>91% • 8%
1	E	235	3%<br>91% • 7%
1	G	235	89% • 9%
2	B	217	96% • •

Continued on next page...

CONFIDENTIAL VALIDATION REPORT

Continued from previous page...

Mol	Chain	Length	Quality of chain
2	D	217	96% • •
2	F	217	% 96% • •
2	H	217	% 95% • •

CONFIDENTIAL VALIDATION REPORT

2 Entry composition (i)

There are 3 unique types of molecules in this entry. The entry contains 13743 atoms, of which 0 are hydrogens and 0 are deuteriums.

In the tables below, the ZeroOcc column contains the number of atoms modelled with zero occupancy, the AltConf column contains the number of residues with at least one atom in alternate conformation and the Trace column contains the number of residues modelled with at most 2 atoms.

- Molecule 1 is a protein called Dupilumab Fab heavy chain.

Mol	Chain	Residues	Atoms					ZeroOcc	AltConf	Trace
			Total	C	N	O	S			
1	A	218	1636	1027	275	327	7	0	4	0
1	C	217	1624	1019	274	324	7	0	3	0
1	E	219	1629	1022	275	324	8	0	1	0
1	G	214	1535	968	257	303	7	0	2	0

- Molecule 2 is a protein called Dupilumab Fab light chain.

Mol	Chain	Residues	Atoms					ZeroOcc	AltConf	Trace
			Total	C	N	O	S			
2	B	215	1681	1058	274	342	7	0	10	0
2	D	214	1648	1039	266	336	7	0	6	0
2	F	214	1636	1030	266	333	7	0	11	0
2	H	212	1563	986	257	313	7	0	2	0

- Molecule 3 is water.

Mol	Chain	Residues	Atoms		ZeroOcc	AltConf
			Total	O		
3	A	140	140	140	0	0
3	B	113	113	113	0	0
3	C	114	114	114	0	0
3	D	72	72	72	0	0

Continued on next page...

CONFIDENTIAL VALIDATION REPORT

Continued from previous page...

Mol	Chain	Residues	Atoms	ZeroOcc	AltConf
3	E	89	Total O<br>89 89	0	0
3	F	94	Total O<br>94 94	0	0
3	G	77	Total O<br>77 77	0	0
3	H	92	Total O<br>92 92	0	0

CONFIDENTIAL VALIDATION REPORT

3 Residue-property plots (i)

These plots are drawn for all protein, RNA and DNA chains in the entry. The first graphic for a chain summarises the proportions of the various outlier classes displayed in the second graphic. The second graphic shows the sequence view annotated by issues in geometry and electron density. Residues are color-coded according to the number of geometric quality criteria for which they contain at least one outlier: green = 0, yellow = 1, orange = 2 and red = 3 or more. A red dot above a residue indicates a poor fit to the electron density (RSRZ > 2). Stretches of 2 or more consecutive residues without any outlier are shown as a green connector. Residues present in the sample, but not in the model, are shown in grey.

• Molecule 1: Dupilumab Fab heavy chain

Chain A:
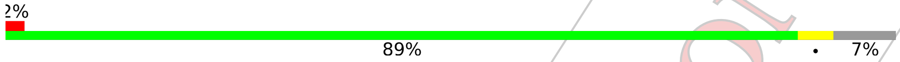

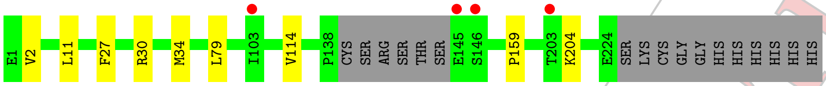

• Molecule 1: Dupilumab Fab heavy chain

Chain C:
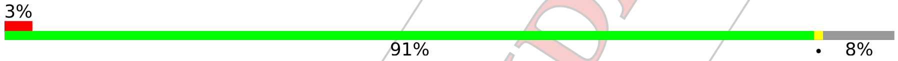

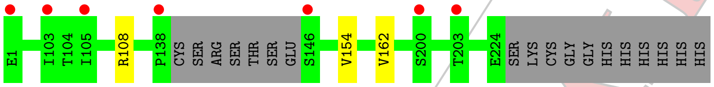

• Molecule 1: Dupilumab Fab heavy chain

Chain E:
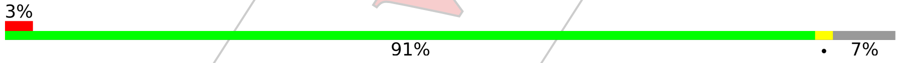

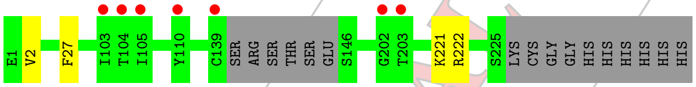

• Molecule 1: Dupilumab Fab heavy chain

Chain G:
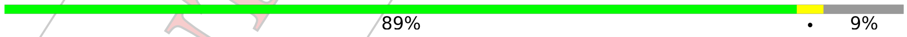

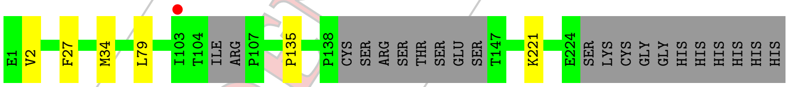

• Molecule 2: Dupilumab Fab light chain

Chain B:
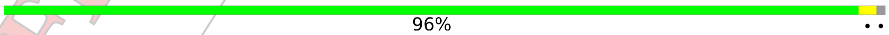

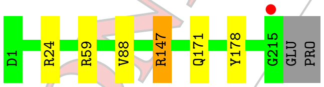

• Molecule 2: Dupilumab Fab light chain

CONFIDENTIAL VALIDATION REPORT

Chain D:

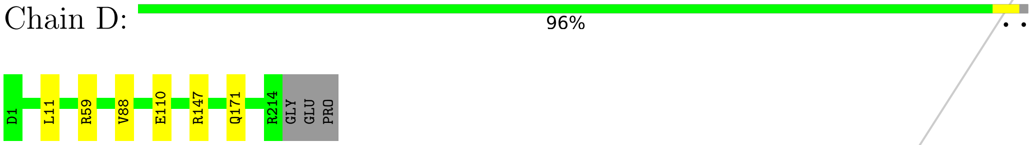

• Molecule 2: Dupilumab Fab light chain

Chain F:

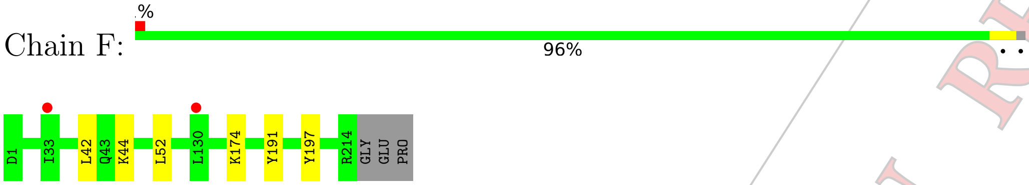

• Molecule 2: Dupilumab Fab light chain

Chain H:

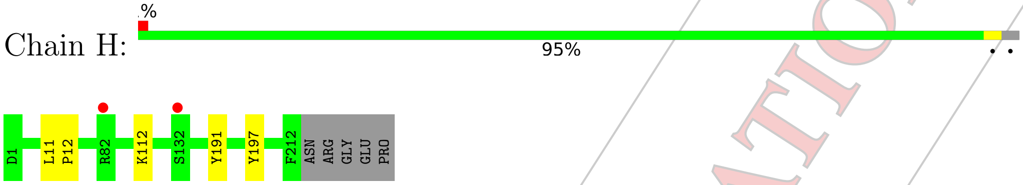

CONFIDENTIAL VALIDATION REPORT

4 Data and refinement statistics (i)

Property	Value	Source
Space group	P 1 21 1	Depositor
Cell constants<br>a, b, c, α, β, γ	109.16Å 79.27Å 109.56Å<br>90.00° 91.87° 90.00°	Depositor
Resolution (Å)	30.00 – 1.62<br>109.50 – 1.62	Depositor<br>EDS
% Data completeness<br>(in resolution range)	95.2 (30.00-1.62)<br>95.2 (109.50-1.62)	Depositor<br>EDS
R_{merge}	0.05	Depositor
R_{sym}	(Not available)	Depositor
$< I/\sigma(I) >$ [1]	2.42 (at 1.62Å)	Xtriage
Refinement program	REFMAC 5.8.0103	Depositor
R, R_{free}	0.210 , 0.236<br>0.210 , 0.237	Depositor<br>DCC
R_{free} test set	11284 reflections (5.01%)	wwPDB-VP
Wilson B-factor (Å^2)	24.8	Xtriage
Anisotropy	0.017	Xtriage
Bulk solvent k_{sol}(e/Å^3), B_{sol}(Å^2)	0.32 , 39.1	EDS
L-test for twinning[2]	$< \|L\| >$ = 0.50, $< L^2 >$ = 0.33	Xtriage
Estimated twinning fraction	0.000 for l,k,-h<br>0.012 for h,-k,-l<br>0.007 for l,-k,h	Xtriage
F_o,F_c correlation	0.96	EDS
Total number of atoms	13743	wwPDB-VP
Average B, all atoms (Å^2)	28.0	wwPDB-VP

Xtriage's analysis on translational NCS is as follows: *The analyses of the Patterson function reveals a significant off-origin peak that is 34.17 % of the origin peak, indicating pseudo-translational symmetry. The chance of finding a peak of this or larger height randomly in a structure without pseudo-translational symmetry is equal to 7.1219e-04. The detected translational NCS is most likely also responsible for the elevated intensity ratio.*

[1] Intensities estimated from amplitudes.
[2] Theoretical values of $< |L| >$, $< L^2 >$ for acentric reflections are 0.5, 0.333 respectively for untwinned datasets, and 0.375, 0.2 for perfectly twinned datasets.

CONFIDENTIAL VALIDATION REPORT

5 Model quality (i)

5.1 Standard geometry (i)

The Z score for a bond length (or angle) is the number of standard deviations the observed value is removed from the expected value. A bond length (or angle) with $|Z| > 5$ is considered an outlier worth inspection. RMSZ is the root-mean-square of all Z scores of the bond lengths (or angles).

Mol	Chain	Bond lengths		Bond angles	
		RMSZ	#$\|Z\|$ >5	RMSZ	#$\|Z\|$ >5
1	A	0.30	0/1684	0.63	0/2293
1	C	0.29	0/1669	0.62	0/2273
1	E	0.29	0/1668	0.61	0/2273
1	G	0.30	0/1575	0.62	0/2152
2	B	0.29	0/1743	0.62	0/2370
2	D	0.28	0/1701	0.61	0/2316
2	F	0.29	0/1698	0.61	0/2316
2	H	0.28	0/1604	0.61	0/2190
All	All	0.29	0/13342	0.62	0/18183

Chiral center outliers are detected by calculating the chiral volume of a chiral center and verifying if the center is modelled as a planar moiety or with the opposite hand.A planarity outlier is detected by checking planarity of atoms in a peptide group, atoms in a mainchain group or atoms of a sidechain that are expected to be planar.

Mol	Chain	#Chirality outliers	#Planarity outliers
1	A	0	1
1	C	0	1
2	B	0	3
2	D	0	2
All	All	0	7

There are no bond length outliers.

There are no bond angle outliers.

There are no chirality outliers.

All (7) planarity outliers are listed below:

Mol	Chain	Res	Type	Group
1	A	30	ARG	Sidechain
2	B	147[A]	ARG	Sidechain
2	B	24	ARG	Sidechain

Continued on next page...

CONFIDENTIAL VALIDATION REPORT

Continued from previous page...

Mol	Chain	Res	Type	Group
2	B	59	ARG	Sidechain
1	C	108	ARG	Sidechain
2	D	147	ARG	Sidechain
2	D	59	ARG	Sidechain

5.2 Too-close contacts (i)

In the following table, the Non-H and H(model) columns list the number of non-hydrogen atoms and hydrogen atoms in the chain respectively. The H(added) column lists the number of hydrogen atoms added and optimized by MolProbity. The Clashes column lists the number of clashes within the asymmetric unit, whereas Symm-Clashes lists symmetry related clashes.

Mol	Chain	Non-H	H(model)	H(added)	Clashes	Symm-Clashes
1	A	1636	0	1589	4	0
1	C	1624	0	1570	1	0
1	E	1629	0	1576	1	0
1	G	1535	0	1435	3	0
2	B	1681	0	1635	5	0
2	D	1648	0	1593	1	0
2	F	1636	0	1545	3	0
2	H	1563	0	1447	2	0
3	A	140	0	0	0	0
3	B	113	0	0	0	0
3	C	114	0	0	0	0
3	D	72	0	0	0	0
3	E	89	0	0	0	0
3	F	94	0	0	0	0
3	G	77	0	0	0	0
3	H	92	0	0	0	0
All	All	13743	0	12390	20	0

The all-atom clashscore is defined as the number of clashes found per 1000 atoms (including hydrogen atoms). The all-atom clashscore for this structure is 1.

All (20) close contacts within the same asymmetric unit are listed below, sorted by their clash magnitude.

Atom-1	Atom-2	Interatomic distance (Å)	Clash overlap (Å)
2:B:147[B]:ARG:HD2	2:B:147[B]:ARG:O	1.66	0.93
2:B:147[B]:ARG:HD2	2:B:147[B]:ARG:C	2.02	0.75
2:D:88:VAL:HG21	2:D:171:GLN:HB3	1.89	0.54

Continued on next page...

CONFIDENTIAL VALIDATION REPORT

Continued from previous page...

Atom-1	Atom-2	Interatomic distance (Å)	Clash overlap (Å)
2:H:12:PRO:HB2	2:H:112:LYS:HE3	1.91	0.53
1:G:135:PRO:HD3	1:G:221:LYS:HE2	1.93	0.49
2:F:42:LEU:HD21	2:F:44:LYS:HE3	1.95	0.47
1:A:2:VAL:HG13	1:A:27:PHE:CD1	2.52	0.45
1:A:11:LEU:HB2	1:A:159:PRO:HG3	1.99	0.44
1:A:34:MET:HB3	1:A:79:LEU:HD22	2.00	0.44
2:B:171:GLN:HG3	2:B:178:TYR:CZ	2.54	0.43
1:C:154:VAL:HG11	1:C:162:VAL:HG11	2.00	0.42
2:H:191:TYR:HA	2:H:197:TYR:OH	2.20	0.42
2:F:191:TYR:HA	2:F:197:TYR:OH	2.20	0.41
2:B:88[B]:VAL:HG21	2:B:171:GLN:HB2	2.01	0.41
2:B:88[A]:VAL:HG21	2:B:171:GLN:HB2	2.02	0.41
1:E:2:VAL:HG13	1:E:27:PHE:CD1	2.56	0.41
2:F:42:LEU:HB2	2:F:52:LEU:HD11	2.03	0.41
1:G:34:MET:HB3	1:G:79:LEU:HD22	2.04	0.40
1:A:2:VAL:HG12	1:A:114:VAL:HG11	2.04	0.40
1:G:2:VAL:HG13	1:G:27:PHE:CD1	2.57	0.40

There are no symmetry-related clashes.

5.3 Torsion angles (i)

5.3.1 Protein backbone (i)

In the following table, the Percentiles column shows the percent Ramachandran outliers of the chain as a percentile score with respect to all X-ray entries followed by that with respect to entries of similar resolution.

The Analysed column shows the number of residues for which the backbone conformation was analysed, and the total number of residues.

Mol	Chain	Analysed	Favoured	Allowed	Outliers	Percentiles	
1	A	218/235 (93%)	215 (99%)	3 (1%)	0	100	100
1	C	216/235 (92%)	209 (97%)	7 (3%)	0	100	100
1	E	216/235 (92%)	213 (99%)	3 (1%)	0	100	100
1	G	210/235 (89%)	206 (98%)	4 (2%)	0	100	100
2	B	223/217 (103%)	218 (98%)	5 (2%)	0	100	100
2	D	218/217 (100%)	214 (98%)	4 (2%)	0	100	100
2	F	223/217 (103%)	217 (97%)	6 (3%)	0	100	100

Continued on next page...

CONFIDENTIAL VALIDATION REPORT

Continued from previous page...

Mol	Chain	Analysed	Favoured	Allowed	Outliers	Percentiles	
2	H	212/217 (98%)	208 (98%)	4 (2%)	0	100	100
All	All	1736/1808 (96%)	1700 (98%)	36 (2%)	0	100	100

There are no Ramachandran outliers to report.

5.3.2 Protein sidechains ⓘ

In the following table, the Percentiles column shows the percent sidechain outliers of the chain as a percentile score with respect to all X-ray entries followed by that with respect to entries of similar resolution.

The Analysed column shows the number of residues for which the sidechain conformation was analysed, and the total number of residues.

Mol	Chain	Analysed	Rotameric	Outliers	Percentiles	
1	A	183/198 (92%)	182 (100%)	1 (0%)	90	82
1	C	180/198 (91%)	180 (100%)	0	100	100
1	E	181/198 (91%)	179 (99%)	2 (1%)	76	58
1	G	160/198 (81%)	160 (100%)	0	100	100
2	B	194/191 (102%)	194 (100%)	0	100	100
2	D	189/191 (99%)	187 (99%)	2 (1%)	76	58
2	F	182/191 (95%)	181 (100%)	1 (0%)	90	82
2	H	165/191 (86%)	164 (99%)	1 (1%)	87	78
All	All	1434/1556 (92%)	1427 (100%)	7 (0%)	90	82

All (7) residues with a non-rotameric sidechain are listed below:

Mol	Chain	Res	Type
1	A	204	LYS
2	D	11	LEU
2	D	110	GLU
1	E	221	LYS
1	E	222	ARG
2	F	174	LYS
2	H	11	LEU

Some sidechains can be flipped to improve hydrogen bonding and reduce clashes. There are no such sidechains identified.

CONFIDENTIAL VALIDATION REPORT

5.3.3 RNA (i)

There are no RNA molecules in this entry.

5.4 Non-standard residues in protein, DNA, RNA chains (i)

There are no non-standard protein/DNA/RNA residues in this entry.

5.5 Carbohydrates (i)

There are no carbohydrates in this entry.

5.6 Ligand geometry (i)

There are no ligands in this entry.

5.7 Other polymers (i)

There are no such residues in this entry.

5.8 Polymer linkage issues (i)

There are no chain breaks in this entry.

CONFIDENTIAL VALIDATION REPORT

6 Fit of model and data (i)

6.1 Protein, DNA and RNA chains (i)

In the following table, the column labelled '#RSRZ> 2' contains the number (and percentage) of RSRZ outliers, followed by percent RSRZ outliers for the chain as percentile scores relative to all X-ray entries and entries of similar resolution. The OWAB column contains the minimum, median, 95th percentile and maximum values of the occupancy-weighted average B-factor per residue. The column labelled 'Q< 0.9' lists the number of (and percentage) of residues with an average occupancy less than 0.9.

Mol	Chain	Analysed	<RSRZ>	#RSRZ>2			OWAB(Å^2)	Q<0.9
1	A	218/235 (92%)	-0.08	4 (1%)	68	67	15, 21, 44, 65	0
1	C	217/235 (92%)	-0.05	7 (3%)	47	44	16, 26, 47, 66	0
1	E	219/235 (93%)	0.01	7 (3%)	47	44	19, 29, 44, 65	0
1	G	214/235 (91%)	-0.05	1 (0%)	90	90	20, 29, 47, 66	0
2	B	215/217 (99%)	-0.15	1 (0%)	90	90	16, 24, 40, 57	0
2	D	214/217 (98%)	-0.13	0	100	100	19, 30, 45, 58	0
2	F	214/217 (98%)	-0.02	2 (0%)	84	84	16, 26, 56, 70	0
2	H	212/217 (97%)	-0.13	2 (0%)	84	84	18, 27, 53, 69	0
All	All	1723/1808 (95%)	-0.07	24 (1%)	75	75	15, 27, 48, 70	0

All (24) RSRZ outliers are listed below:

Mol	Chain	Res	Type	RSRZ
2	B	215	GLY	5.4
1	A	145	GLU	3.8
1	A	103	ILE	3.7
1	C	138	PRO	3.2
1	E	104	THR	3.1
1	C	203	THR	3.0
1	C	103	ILE	3.0
1	C	1	GLU	2.9
1	E	203	THR	2.9
2	F	130	LEU	2.8
1	G	103	ILE	2.6
1	A	146	SER	2.6
1	C	200	SER	2.5
2	F	33	ILE	2.4
1	E	105	ILE	2.4
1	C	146	SER	2.4

Continued on next page...

CONFIDENTIAL VALIDATION REPORT

Continued from previous page...

Mol	Chain	Res	Type	RSRZ
1	C	105	ILE	2.3
1	E	110	TYR	2.3
1	E	103	ILE	2.2
2	H	132	SER	2.2
1	A	203	THR	2.2
1	E	202	GLY	2.1
2	H	82	ARG	2.1
1	E	139	CYS	2.1

6.2 Non-standard residues in protein, DNA, RNA chains (i)

There are no non-standard protein/DNA/RNA residues in this entry.

6.3 Carbohydrates (i)

There are no carbohydrates in this entry.

6.4 Ligands (i)

There are no ligands in this entry.

6.5 Other polymers (i)

There are no such residues in this entry.

CONFIDENTIAL VALIDATION REPORT
