# Supplementary material for: Rapid and robust antibody Fab fragment crystallization utilizing edge-to-edge beta-sheet packing
Source: PLoS One. 2020 Sep 11;15(9):e0232311. doi: 10.1371/journal.pone.0232311 (PMC7485759; doi:10.1371/journal.pone.0232311)
Supplement: S7 File — (PDF) [file pone.0232311.s010.pdf]

Full wwPDB X-ray Structure Validation Report (i)

CONFIDENTIAL VALIDATION REPORT

Apr 13, 2020 – 11:47 AM EDT

PDB ID	:	6WIR
Title	:	Fab antigen complex
Deposited on	:	2020-04-10
Resolution	:	2.96 Å(reported)

This is a Full wwPDB X-ray Structure Validation Report.

This report is produced by the wwPDB biocuration pipeline after annotation of the structure.

We welcome your comments at *validation@mail.wwpdb.org*
A user guide is available at
https://www.wwpdb.org/validation/2017/XrayValidationReportHelp
with specific help available everywhere you see the (i) symbol.

The following versions of software and data (see references (i)) were used in the production of this report:

MolProbity	:	4.02b-467
Xtriage (Phenix)	:	1.13
EDS	:	2.10.1
Percentile statistics	:	20171227.v01 (using entries in the PDB archive December 27th 2017)
Refmac	:	5.8.0158
CCP4	:	7.0.044 (Gargrove)
Ideal geometry (proteins)	:	Engh & Huber (2001)
Ideal geometry (DNA, RNA)	:	Parkinson et al. (1996)
Validation Pipeline (wwPDB-VP)	:	2.10.1

1 Overall quality at a glance (i)

The following experimental techniques were used to determine the structure:
X-RAY DIFFRACTION

The reported resolution of this entry is 2.96 Å.

Percentile scores (ranging between 0-100) for global validation metrics of the entry are shown in the following graphic. The table shows the number of entries on which the scores are based.

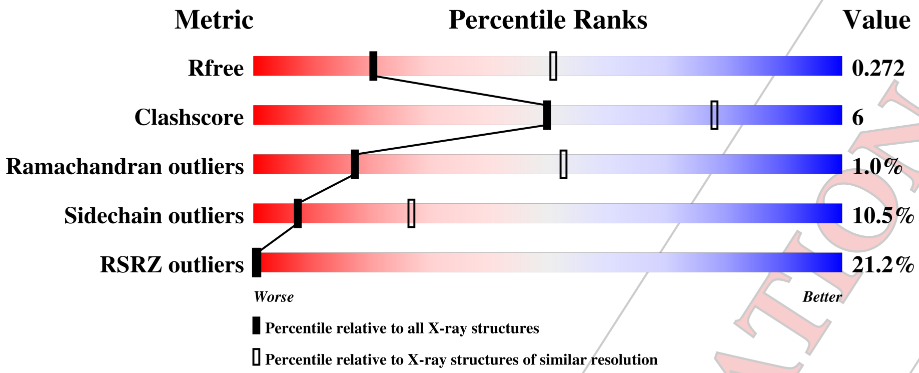

Metric	Whole archive (#Entries)	Similar resolution (#Entries, resolution range(Å))
R_{free}	111664	2641 (3.00-2.92)
Clashscore	122126	2988 (3.00-2.92)
Ramachandran outliers	120053	2892 (3.00-2.92)
Sidechain outliers	120020	2895 (3.00-2.92)
RSRZ outliers	108989	2527 (3.00-2.92)

The table below summarises the geometric issues observed across the polymeric chains and their fit to the electron density. The red, orange, yellow and green segments on the lower bar indicate the fraction of residues that contain outliers for >=3, 2, 1 and 0 types of geometric quality criteria respectively. A grey segment represents the fraction of residues that are not modelled. The numeric value for each fraction is indicated below the corresponding segment, with a dot representing fractions <=5% The upper red bar (where present) indicates the fraction of residues that have poor fit to the electron density. The numeric value is given above the bar.

Mol	Chain	Length	Quality of chain
1	A	236	17% / 74% 22% •
2	B	213	13% / 71% 28%
3	C	161	29% / 43% 11% • 45%

CONFIDENTIAL VALIDATION REPORT

2 Entry composition (i)

There are 4 unique types of molecules in this entry. The entry contains 4125 atoms, of which 0 are hydrogens and 0 are deuteriums.

In the tables below, the ZeroOcc column contains the number of atoms modelled with zero occupancy, the AltConf column contains the number of residues with at least one atom in alternate conformation and the Trace column contains the number of residues modelled with at most 2 atoms.

- Molecule 1 is a protein called Fab heavy chain.

Mol	Chain	Residues	Atoms	ZeroOcc	AltConf	Trace
1	A	227	Total 1721, C 1095, N 288, O 331, S 7	0	0	0

- Molecule 2 is a protein called Fab light chain.

Mol	Chain	Residues	Atoms	ZeroOcc	AltConf	Trace
2	B	212	Total 1610, C 1002, N 275, O 328, S 5	0	0	0

- Molecule 3 is a protein called Interleukin-17A.

Mol	Chain	Residues	Atoms	ZeroOcc	AltConf	Trace
3	C	89	Total 722, C 452, N 133, O 131, S 6	0	0	0

There are 6 discrepancies between the modelled and reference sequences:

Chain	Residue	Modelled	Actual	Comment	Reference
C	133	HIS	-	expression tag	UNP Q16552
C	134	HIS	-	expression tag	UNP Q16552
C	135	HIS	-	expression tag	UNP Q16552
C	136	HIS	-	expression tag	UNP Q16552
C	137	HIS	-	expression tag	UNP Q16552
C	138	HIS	-	expression tag	UNP Q16552

- Molecule 4 is water.

Mol	Chain	Residues	Atoms	ZeroOcc	AltConf
4	A	34	Total 34, O 34	0	0
4	B	36	Total 36, O 36	0	0

Continued on next page...

CONFIDENTIAL VALIDATION REPORT

Continued from previous page...

Mol	Chain	Residues	Atoms	ZeroOcc	AltConf
4	C	2	Total O<br>2 2	0	0

CONFIDENTIAL VALIDATION REPORT

3 Residue-property plots (i)

These plots are drawn for all protein, RNA and DNA chains in the entry. The first graphic for a chain summarises the proportions of the various outlier classes displayed in the second graphic. The second graphic shows the sequence view annotated by issues in geometry and electron density. Residues are color-coded according to the number of geometric quality criteria for which they contain at least one outlier: green = 0, yellow = 1, orange = 2 and red = 3 or more. A red dot above a residue indicates a poor fit to the electron density (RSRZ > 2). Stretches of 2 or more consecutive residues without any outlier are shown as a green connector. Residues present in the sample, but not in the model, are shown in grey.

- Molecule 1: Fab heavy chain

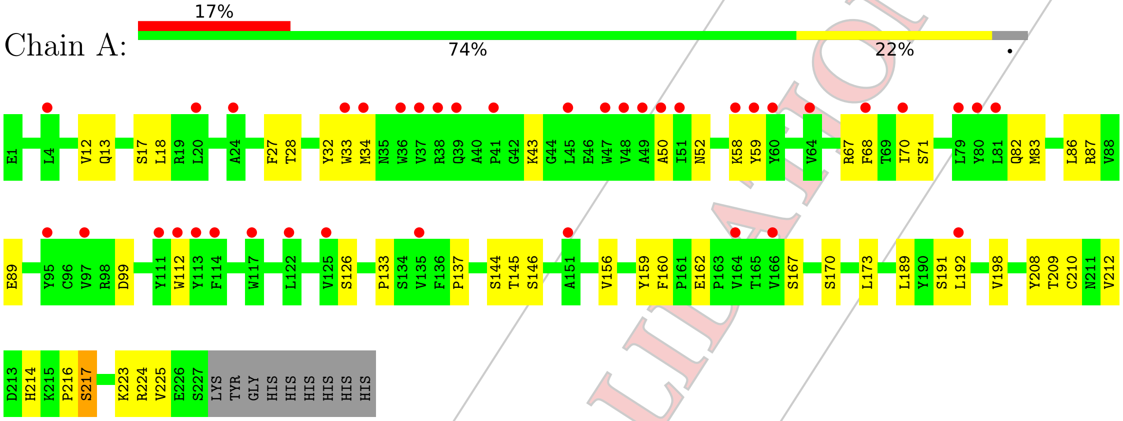

- Molecule 2: Fab light chain

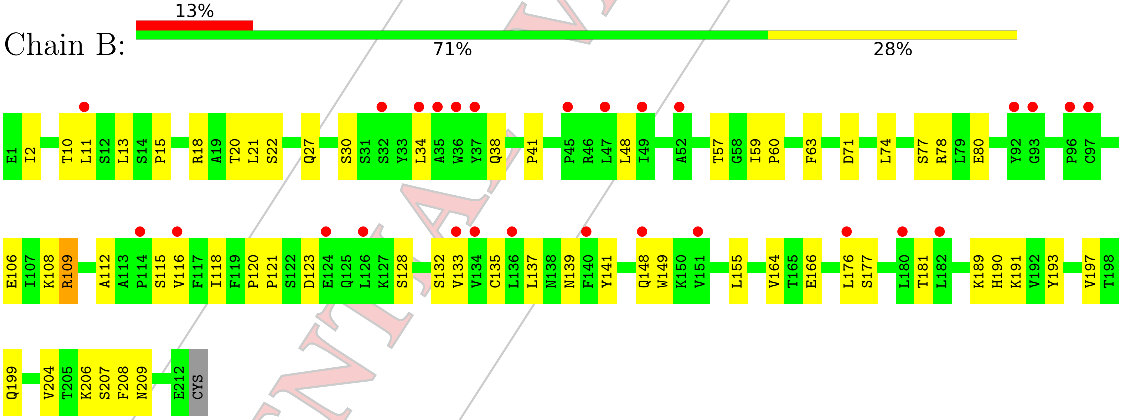

- Molecule 3: Interleukin-17A

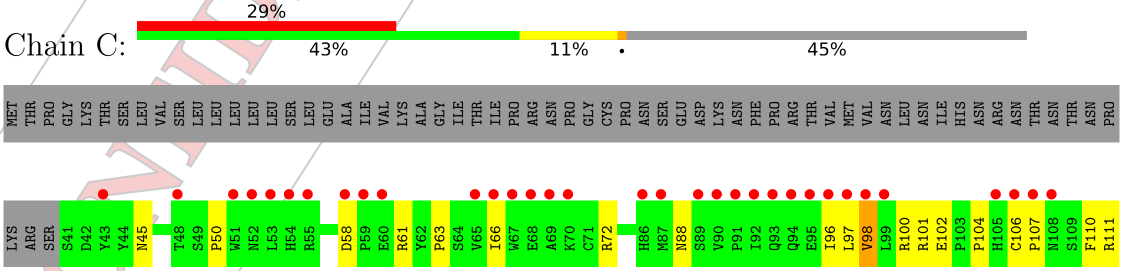

CONFIDENTIAL VALIDATION REPORT

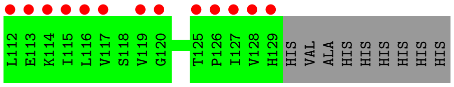

CONFIDENTIAL VALIDATION REPORT

4 Data and refinement statistics (i)

Property	Value	Source
Space group	P 41 21 2	Depositor
Cell constants<br>a, b, c, α, β, γ	73.40Å 73.40Å 326.41Å<br>90.00° 90.00° 90.00°	Depositor
Resolution (Å)	71.61 – 2.96<br>71.61 – 2.96	Depositor<br>EDS
% Data completeness<br>(in resolution range)	100.0 (71.61-2.96)<br>100.0 (71.61-2.96)	Depositor<br>EDS
R_{merge}	0.08	Depositor
R_{sym}	(Not available)	Depositor
$< I/\sigma(I) >$ [1]	2.39 (at 2.96Å)	Xtriage
Refinement program	BUSTER 2.11.7	Depositor
R, R_{free}	0.217 , 0.260<br>0.227 , 0.272	Depositor<br>DCC
R_{free} test set	977 reflections (4.96%)	wwPDB-VP
Wilson B-factor (Å^2)	94.3	Xtriage
Anisotropy	0.043	Xtriage
Bulk solvent k_{sol}(e/Å^3), B_{sol}(Å^2)	0.32 , 91.3	EDS
L-test for twinning[2]	$< \|L\| > = 0.49$, $< L^2 > = 0.32$	Xtriage
Estimated twinning fraction	No twinning to report.	Xtriage
F_o,F_c correlation	0.91	EDS
Total number of atoms	4125	wwPDB-VP
Average B, all atoms (Å^2)	111.0	wwPDB-VP

Xtriage's analysis on translational NCS is as follows: *The largest off-origin peak in the Patterson function is 4.48% of the height of the origin peak. No significant pseudotranslation is detected.*

[1]Intensities estimated from amplitudes.

[2]Theoretical values of $< |L| >$, $< L^2 >$ for acentric reflections are 0.5, 0.333 respectively for untwinned datasets, and 0.375, 0.2 for perfectly twinned datasets.

CONFIDENTIAL VALIDATION REPORT

5 Model quality (i)

5.1 Standard geometry (i)

The Z score for a bond length (or angle) is the number of standard deviations the observed value is removed from the expected value. A bond length (or angle) with $|Z| > 5$ is considered an outlier worth inspection. RMSZ is the root-mean-square of all Z scores of the bond lengths (or angles).

Mol	Chain	Bond lengths		Bond angles	
		RMSZ	#$|Z|$ >5	RMSZ	#$|Z|$ >5
1	A	0.54	0/1766	0.79	0/2411
2	B	0.54	0/1643	0.76	0/2230
3	C	0.48	0/743	0.68	0/1015
All	All	0.53	0/4152	0.76	0/5656

There are no bond length outliers.

There are no bond angle outliers.

There are no chirality outliers.

There are no planarity outliers.

5.2 Too-close contacts (i)

In the following table, the Non-H and H(model) columns list the number of non-hydrogen atoms and hydrogen atoms in the chain respectively. The H(added) column lists the number of hydrogen atoms added and optimized by MolProbity. The Clashes column lists the number of clashes within the asymmetric unit, whereas Symm-Clashes lists symmetry related clashes.

Mol	Chain	Non-H	H(model)	H(added)	Clashes	Symm-Clashes
1	A	1721	0	1644	20	0
2	B	1610	0	1550	23	0
3	C	722	0	688	7	0
4	A	34	0	0	1	0
4	B	36	0	0	0	0
4	C	2	0	0	0	0
All	All	4125	0	3882	50	0

The all-atom clashscore is defined as the number of clashes found per 1000 atoms (including hydrogen atoms). The all-atom clashscore for this structure is 6.

All (50) close contacts within the same asymmetric unit are listed below, sorted by their clash magnitude.

CONFIDENTIAL VALIDATION REPORT

Atom-1	Atom-2	Interatomic distance (Å)	Clash overlap (Å)
2:B:38:GLN:HB2	2:B:48:LEU:HD11	1.60	0.83
2:B:18:ARG:HG3	2:B:77:SER:HA	1.69	0.73
1:A:67:ARG:HH12	1:A:87:ARG:HG3	1.55	0.71
2:B:164:VAL:HG22	2:B:176:LEU:HD12	1.74	0.69
3:C:101:ARG:HD3	3:C:106:CYS:H	1.57	0.68
1:A:12:VAL:HG21	1:A:18:LEU:HG	1.74	0.68
2:B:109:ARG:NH2	2:B:112:ALA:HB2	2.12	0.64
1:A:160:PHE:HB2	1:A:189:LEU:HD23	1.80	0.63
2:B:108:LYS:HA	2:B:141:TYR:OH	2.02	0.60
3:C:63:PRO:HD2	3:C:98:VAL:HG22	1.85	0.58
1:A:50:ALA:HB3	1:A:59:TYR:HB2	1.86	0.57
1:A:214:HIS:CE1	1:A:217:SER:HB3	2.41	0.55
2:B:41:PRO:HG3	2:B:166:GLU:HG2	1.88	0.55
1:A:214:HIS:CD2	1:A:216:PRO:HD2	2.42	0.54
1:A:58:LYS:HE3	1:A:70:ILE:O	2.07	0.54
2:B:191:LYS:O	2:B:209:ASN:HA	2.07	0.54
2:B:164:VAL:CG2	2:B:176:LEU:HD12	2.36	0.54
2:B:148:GLN:HB3	2:B:155:LEU:HD11	1.90	0.52
1:A:83:MET:HB3	1:A:86:LEU:HD21	1.93	0.51
2:B:21:LEU:HD22	2:B:74:LEU:HD23	1.93	0.51
1:A:156:VAL:HG13	1:A:212:VAL:HG11	1.94	0.49
2:B:109:ARG:HH22	2:B:112:ALA:HB2	1.77	0.49
2:B:2:ILE:HG12	2:B:27:GLN:HB2	1.95	0.48
1:A:32:TYR:HB2	1:A:34:MET:HE2	1.95	0.48
3:C:50:PRO:HB2	3:C:72:ARG:HH11	1.80	0.47
1:A:133:PRO:HB3	1:A:159:TYR:HB3	1.97	0.47
1:A:17:SER:HA	1:A:83:MET:O	2.15	0.46
2:B:116:VAL:HG12	2:B:206:LYS:HG3	1.97	0.46
1:A:214:HIS:ND1	1:A:217:SER:HB3	2.30	0.45
1:A:137:PRO:HB2	1:A:225:VAL:HG13	1.98	0.45
3:C:96:ILE:HG13	3:C:97:LEU:H	1.81	0.45
2:B:120:PRO:HB3	2:B:208:PHE:CZ	2.52	0.44
2:B:190:HIS:HB2	2:B:193:TYR:OH	2.18	0.44
2:B:48:LEU:HD23	2:B:59:ILE:HD12	2.00	0.43
3:C:50:PRO:HB2	3:C:72:ARG:HD2	2.01	0.43
3:C:58:ASP:HB3	3:C:61:ARG:HB2	1.99	0.43
1:A:198:VAL:HG11	1:A:208:TYR:CE1	2.55	0.42
1:A:67:ARG:NH1	1:A:87:ARG:HG3	2.29	0.42
2:B:60:PRO:HG2	2:B:63:PHE:HD1	1.83	0.42
3:C:101:ARG:HG3	3:C:104:PRO:HA	2.02	0.42
2:B:137:LEU:HD11	2:B:197:VAL:CG2	2.50	0.42
2:B:11:LEU:HG	2:B:13:LEU:HD13	2.02	0.42

Continued on next page...

CONFIDENTIAL VALIDATION REPORT

Continued from previous page...

Atom-1	Atom-2	Interatomic distance (Å)	Clash overlap (Å)
1:A:68:PHE:HA	1:A:82:GLN:O	2.21	0.41
2:B:121:PRO:HB3	2:B:132:SER:H	1.85	0.41
2:B:137:LEU:HD22	2:B:176:LEU:HD22	2.03	0.41
2:B:118:ILE:HG22	2:B:206:LYS:HB3	2.03	0.41
2:B:135:CYS:HB2	2:B:149:TRP:CH2	2.56	0.41
1:A:27:PHE:HB2	4:A:317:HOH:O	2.21	0.41
1:A:209:THR:HA	1:A:223:LYS:O	2.21	0.41
1:A:33:TRP:HB2	1:A:99:ASP:HB2	2.03	0.40

There are no symmetry-related clashes.

5.3 Torsion angles (i)

5.3.1 Protein backbone (i)

In the following table, the Percentiles column shows the percent Ramachandran outliers of the chain as a percentile score with respect to all X-ray entries followed by that with respect to entries of similar resolution.

The Analysed column shows the number of residues for which the backbone conformation was analysed, and the total number of residues.

Mol	Chain	Analysed	Favoured	Allowed	Outliers	Percentiles	
1	A	225/236 (95%)	201 (89%)	21 (9%)	3 (1%)	13	45
2	B	210/213 (99%)	196 (93%)	13 (6%)	1 (0%)	31	69
3	C	87/161 (54%)	76 (87%)	10 (12%)	1 (1%)	16	50
All	All	522/610 (86%)	473 (91%)	44 (8%)	5 (1%)	17	53

All (5) Ramachandran outliers are listed below:

Mol	Chain	Res	Type
1	A	146	SER
2	B	139	ASN
1	A	145	THR
1	A	144	SER
3	C	107	PRO

CONFIDENTIAL VALIDATION REPORT

5.3.2 Protein sidechains (i)

In the following table, the Percentiles column shows the percent sidechain outliers of the chain as a percentile score with respect to all X-ray entries followed by that with respect to entries of similar resolution.

The Analysed column shows the number of residues for which the sidechain conformation was analysed, and the total number of residues.

Mol	Chain	Analysed	Rotameric	Outliers	Percentiles	
1	A	186/203 (92%)	169 (91%)	17 (9%)	10	34
2	B	179/184 (97%)	157 (88%)	22 (12%)	5	20
3	C	83/150 (55%)	75 (90%)	8 (10%)	9	32
All	All	448/537 (83%)	401 (90%)	47 (10%)	7	27

All (47) residues with a non-rotameric sidechain are listed below:

Mol	Chain	Res	Type
1	A	13	GLN
1	A	28	THR
1	A	43	LYS
1	A	52	ASN
1	A	71	SER
1	A	89	GLU
1	A	112	TRP
1	A	126	SER
1	A	162	GLU
1	A	167	SER
1	A	170	SER
1	A	173	LEU
1	A	191	SER
1	A	192	LEU
1	A	210	CYS
1	A	217	SER
1	A	224	ARG
2	B	10	THR
2	B	15	PRO
2	B	20	THR
2	B	22	SER
2	B	30	SER
2	B	34	LEU
2	B	57	THR
2	B	71	ASP
2	B	78	ARG

Continued on next page...

CONFIDENTIAL VALIDATION REPORT

Continued from previous page...

Mol	Chain	Res	Type
2	B	80	GLU
2	B	106	GLU
2	B	109	ARG
2	B	115	SER
2	B	123	ASP
2	B	128	SER
2	B	133	VAL
2	B	177	SER
2	B	181	THR
2	B	189	LYS
2	B	199	GLN
2	B	204	VAL
2	B	207	SER
3	C	45	ASN
3	C	66	ILE
3	C	88	ASN
3	C	98	VAL
3	C	100	ARG
3	C	102	GLU
3	C	110	PHE
3	C	111	ARG

Some sidechains can be flipped to improve hydrogen bonding and reduce clashes. All (4) such sidechains are listed below:

Mol	Chain	Res	Type
1	A	178	HIS
2	B	27	GLN
2	B	138	ASN
3	C	45	ASN

5.3.3 RNA (i)

There are no RNA molecules in this entry.

5.4 Non-standard residues in protein, DNA, RNA chains (i)

There are no non-standard protein/DNA/RNA residues in this entry.

CONFIDENTIAL VALIDATION REPORT

5.5 Carbohydrates (i)

There are no carbohydrates in this entry.

5.6 Ligand geometry (i)

There are no ligands in this entry.

5.7 Other polymers (i)

There are no such residues in this entry.

5.8 Polymer linkage issues (i)

There are no chain breaks in this entry.

CONFIDENTIAL VALIDATION REPORT

6 Fit of model and data (i)

6.1 Protein, DNA and RNA chains (i)

In the following table, the column labelled '#RSRZ> 2' contains the number (and percentage) of RSRZ outliers, followed by percent RSRZ outliers for the chain as percentile scores relative to all X-ray entries and entries of similar resolution. The OWAB column contains the minimum, median, 95th percentile and maximum values of the occupancy-weighted average B-factor per residue. The column labelled 'Q< 0.9' lists the number of (and percentage) of residues with an average occupancy less than 0.9.

Mol	Chain	Analysed	<RSRZ>	#RSRZ>2			OWAB(Å^2)	Q<0.9
1	A	227/236 (96%)	0.99	39 (17%)	1	1	68, 99, 130, 145	0
2	B	212/213 (99%)	0.79	27 (12%)	3	2	71, 100, 125, 145	0
3	C	89/161 (55%)	2.54	46 (51%)	0	0	97, 132, 244, 259	0
All	All	528/610 (86%)	1.17	112 (21%)	1	0	68, 103, 190, 259	0

All (112) RSRZ outliers are listed below:

Mol	Chain	Res	Type	RSRZ
3	C	112	LEU	7.5
3	C	67	TRP	7.4
3	C	93	GLN	7.4
3	C	115	ILE	7.3
3	C	116	LEU	7.1
3	C	65	VAL	6.9
3	C	96	ILE	6.6
3	C	53	LEU	5.9
3	C	97	LEU	5.8
3	C	120	GLY	5.7
3	C	59	PRO	5.6
3	C	92	ILE	5.6
3	C	90	VAL	5.5
3	C	113	GLU	5.4
3	C	98	VAL	5.2
3	C	70	LYS	5.2
1	A	112	TRP	4.9
3	C	119	VAL	4.8
2	B	37	TYR	4.7
1	A	50	ALA	4.6
3	C	55	ARG	4.6
1	A	97	VAL	4.3
1	A	48	VAL	4.1

Continued on next page...

CONFIDENTIAL VALIDATION REPORT

Continued from previous page...

Mol	Chain	Res	Type	RSRZ
3	C	94	GLN	4.1
1	A	36	TRP	3.9
3	C	129	HIS	3.8
1	A	34	MET	3.7
3	C	66	ILE	3.7
1	A	37	VAL	3.7
2	B	47	LEU	3.7
3	C	114	LYS	3.6
3	C	99	LEU	3.6
3	C	68	GLU	3.5
1	A	47	TRP	3.5
3	C	89	SER	3.5
1	A	41	PRO	3.5
1	A	33	TRP	3.4
1	A	114	PHE	3.4
1	A	20	LEU	3.4
3	C	117	VAL	3.4
1	A	79	LEU	3.4
1	A	111	TYR	3.3
2	B	49	ILE	3.2
3	C	43	TYR	3.2
2	B	126	LEU	3.2
2	B	97	CYS	3.1
2	B	92	TYR	3.1
3	C	58	ASP	3.1
3	C	48	THR	3.0
1	A	58	LYS	3.0
3	C	107	PRO	3.0
3	C	128	VAL	2.9
3	C	69	ALA	2.9
1	A	122	LEU	2.9
3	C	91	PRO	2.9
1	A	4	LEU	2.9
1	A	81	LEU	2.9
3	C	60	GLU	2.9
2	B	116	VAL	2.9
2	B	35	ALA	2.9
3	C	105	HIS	2.9
1	A	95	TYR	2.8
1	A	70	ILE	2.8
2	B	36	TRP	2.8
3	C	126	PRO	2.8

Continued on next page...

CONFIDENTIAL VALIDATION REPORT

Continued from previous page...

Mol	Chain	Res	Type	RSRZ
1	A	117	TRP	2.7
1	A	45	LEU	2.7
2	B	180	LEU	2.7
2	B	140	PHE	2.6
3	C	127	ILE	2.6
2	B	148	GLN	2.5
1	A	113	TYR	2.5
1	A	49	ALA	2.5
2	B	45	PRO	2.5
1	A	60	TYR	2.5
2	B	182	LEU	2.5
3	C	87	MET	2.5
2	B	136	LEU	2.5
1	A	38	ARG	2.5
3	C	51	TRP	2.5
1	A	64	VAL	2.4
3	C	125	THR	2.4
2	B	124	GLU	2.4
3	C	86	HIS	2.4
1	A	166	VAL	2.4
1	A	59	TYR	2.3
2	B	114	PRO	2.3
1	A	51	ILE	2.3
1	A	68	PHE	2.3
3	C	52	ASN	2.3
1	A	80	TYR	2.3
2	B	32	SER	2.2
2	B	34	LEU	2.2
2	B	133	VAL	2.2
2	B	11	LEU	2.2
2	B	134	VAL	2.2
3	C	106	CYS	2.2
3	C	54	HIS	2.2
1	A	164	VAL	2.1
3	C	108	ASN	2.1
1	A	24	ALA	2.1
3	C	95	GLU	2.1
1	A	39	GLN	2.1
1	A	135	VAL	2.1
2	B	52	ALA	2.1
2	B	151	VAL	2.1
2	B	93	GLY	2.1

Continued on next page...

CONFIDENTIAL VALIDATION REPORT

Continued from previous page...

Mol	Chain	Res	Type	RSRZ
2	B	176	LEU	2.0
1	A	151	ALA	2.0
2	B	96	PRO	2.0
1	A	192	LEU	2.0
1	A	125	VAL	2.0

6.2 Non-standard residues in protein, DNA, RNA chains (i)

There are no non-standard protein/DNA/RNA residues in this entry.

6.3 Carbohydrates (i)

There are no carbohydrates in this entry.

6.4 Ligands (i)

There are no ligands in this entry.

6.5 Other polymers (i)

There are no such residues in this entry.

CONFIDENTIAL VALIDATION REPORT
